# Osteogenic and Anti-Inflammatory Behavior of Injectable Calcium Phosphate Loaded with Therapeutic Drugs

**DOI:** 10.3390/nano10091743

**Published:** 2020-09-03

**Authors:** Ines Fasolino, Alessandra Soriente, Luigi Ambrosio, Maria Grazia Raucci

**Affiliations:** Institute of Polymers, Composites and Biomaterials—National Research Council (IPCB-CNR), Mostra d’Oltremare pad.20—Viale J.F. Kennedy 54, 80125 Naples, Italy; alessandra.soriente@cnr.it (A.S.); luigi.ambrosio@cnr.it (L.A.)

**Keywords:** injectable biomaterials, bone tissue regeneration, calcium phosphates, inflammation treatment, drug loaded biomaterials, sol-gel method and in vitro model

## Abstract

Bone fractures related to musculoskeletal disorders determine long-term disability in older people with a consequent significant economic burden. The recovery of pathologically impaired tissue architecture allows avoiding bone loss-derived consequences such as bone height reduction, deterioration of bone structure, inflamed bone pain, and high mortality for thighbone fractures. Actually, standard therapy for osteoporosis treatment is based on the systemic administration of biphosphonates and anti-inflammatory drugs, which entail several side effects including gastrointestinal (GI) diseases, fever, and articular pain. Hence, the demand of innovative therapeutic approaches for locally treating bone lesions has been increasing in the last few years. In this scenario, the development of injectable materials loaded with therapeutically active agents (i.e., anti-inflammatory drugs, antibiotics, and peptides mimicking growth factors) could be an effective tool to treat bone loss and inflammation related to musculoskeletal diseases, including osteoporosis and osteoarthritis. According to this challenge, here, we propose three different compositions of injectable calcium phosphates (CaP) as new carrier materials of therapeutic compounds such as bisphosphonates (i.e., alendronate), anti-inflammatory drugs (i.e., diclofenac sodium), and natural molecules (i.e., harpagoside) for the local bone disease treatment. Biological quantitative analyses were performed for screening osteoinductive and anti-inflammatory properties of injectable drug-loaded systems. Meanwhile, cell morphological features were analyzed through scanning electron microscopy and confocal investigations. The results exhibited that the three systems exerted an osteoinductive effect during later phases of osteogenesis. Simultaneously, all compositions showed an anti-inflammatory activity on inflammation in vitro models.

## 1. Introduction

Bone disorders affect most mammalian species and dramatically influence the economy and healthcare costs of countries at higher incidence. Notably, osteoporosis is marked by a deficiency in bone remodeling in favor of osteocatabolic processes, causing a decrease of trabecular mass density. Moreover, the concurrent inflammatory reaction increases fracture risk with a higher mortality ratio (10–20%) especially due to proximal femur breaking [1]. Hence, a peculiar spotlight is placed on inflammatory mechanisms involved in musculoskeletal disorders. Most in vitro models have tested the amount of inflammatory cytokines to identify innovative strategies for treating inflammatory bone injuries and preventing fractures [2]. Nowadays, conventional osteoporosis therapies are based on the systemic administration of biphosphonates, anti-inflammatory drugs, and selective estrogen receptor modulators. Bisphosphonates (BPs) are selectively taken up by osteoclasts and strongly arrest bone resorption by inducing osteoclast apoptosis [3]. BPs are active in the therapy of several diseases such as osteoporosis, Paget’s disease, multiple myeloma, hypercalcemia of malignancy, and osteolytic lesions of cancer metastasis. However, a long-term therapy with BPs causes jaw osteonecrosis in many patients. Furthermore, a continuing exposure to BPs is correlated with an expanded risk of developing a wide range of adverse effects such as gastrointestinal (GI), fever, and articular pain. To overtake these inconveniences related to the systemic administration of bisphosphonates, innovative approaches have been undertaken to treat osteoporosis. Until now, bone grafting is the landmark to generate bone healing in fracture defects in an osteoporotic bone tissue [4,5]. Recently, novel approaches suggest the implantation of bioactivated calcium phosphate cements with osteoinductive agents to enhance new bone tissue formation. In this context, injectable calcium phosphate-based materials showed several advantages concerning biocompatibility, bioactivity, and osteoconductive properties. Previous studies established that calcium phosphate-based materials (CaPs) possess a good injectability, thus satisfying the rheological properties required for minimally invasive surgical procedures [6]. In addition, these materials may be used as a drug-delivery system owing to their good physical and chemical features [7]. Hence, several drugs useful in the osteoporosis treatment (i.e., bisphosphonates) could be loaded in CaPs in order to realize a local delivery of agents in bone defect. Thus, scaffolds for bone tissue regeneration could also be used as devices for the local release of synthetic (i.e., diclofenac sodium) or natural anti-inflammatory compounds. One such class of natural compounds is the iridoids such as harpagoside (HR), which is a natural anti-inflammatory component isolated from Harpagophytum procumbens (devil’s claw). Since ancient times, extracts of the secondary roots of Harpagophytum have showed potent anti-inflammatory properties without significant side effects [8]. Later, HR anti-inflammatory benefits were confirmed in clinical trials that have shown no toxicity and a good tolerability of HR-based products [9]. HR anti-inflammatory potential is related to its capability to inhibit COX-1 and COX-2 (cyclooxygenases 1 and 2), which are enzymes involved in inflammatory mediator’s synthesis [10]. In addition, it has been demonstrated that HR also inhibited NO (nitric oxide), interleukins 1β and 6 (IL-1β and IL-6), and tumor necrosis factor-α (TNF-α) production [11]. The ability to block high IL-1β levels makes HR a useful candidate for osteoarthritis treatment, because in chondrocytes, high values of IL-1β are implicated in the osteoarthritis pathogenesis [12]. The anti-inflammatory potential of HR has not been widely investigated in the field of bone repair. Indeed, only one study reported that HR causes an in vitro inhibition of receptor activator of nuclear factor κB ligand (RANKL)-induced osteoclastogenesis and in vivo suppression inflammation-induced of bone loss [13]. Herein, our objective was to create therapeutic injectable materials to locally treat bone diseases. The CaP-based systems were fabricated through the sol-gel method, which allows obtaining full injectability and nano-hydroxyapatite with low crystallinity mimicking natural bone mineral components [14]. Furthermore, this technology allows a loading of thermo-sensible drugs in the variously shaped materials and to synthesize organic-inorganic hybrid materials [15,16,17,18]. In detail, this work aims to examine the effects of synthetic drugs (i.e., alendronate, diclofenac sodium) and natural anti-inflammatory molecules (i.e., harpagoside) loaded injectable CaP-based carriers for locally treating bone lesions. To this end, the effect of these therapeutic materials in terms of osteoinductive and anti-inflammatory potential was evaluated.

## 2. Materials and Methods

### 2.1. Biomaterials Synthesis

The calcium phosphate materials (CaPs) were synthesized at room temperature by sol–gel technology as reported in a previous study [14]. Briefly, CaP were obtained by using calcium nitrate tetrahydrate (Ca(NO_3_)_2_·4H_2_O, 99% A.C.S. reagent, Sigma-Aldrich, Milan, Italy) and ammonium phosphate dibasic ((NH_4_)_2_HPO_4_, (A.C.S. reagent, Aldrich) as precursors of Ca^2+^ and PO_4_^3−^ ions, respectively [14]. For the synthesis of hydroxyapatite, a molar ratio of about 1.67 between Ca and P was used.

In brief, the procedure included the preparation and mixing of a 3.58 mM (NH_4_)_2_HPO_4_ solution to a 3.00 mM Ca solution under stirring at 200 rpm and 40 °C until gelation occurred. The medium alkalinity was adjusted by adding ammonium hydroxide (NH_4_OH) up to pH 9. After gelling, the gels were dialyzed in 0.01 M phosphate-buffered saline (PBS), pH 7.4, until equilibrated to the buffer pH.

**Figure 1:**
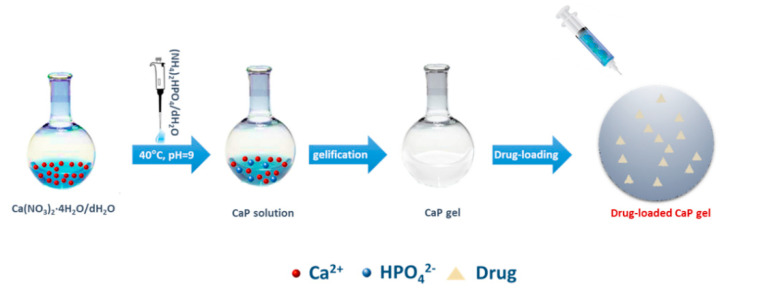
Scheme representing the preparation of drug-loaded calcium phosphate-based (CaP) injectable materials. The drug represents alendronate or diclofenac or harpagoside.

The sterilization of CaPs was achieved by using UV irradiation for 1 h. Meanwhile, drugs such as alendronate (Ale, 1 μM), diclofenac sodium (Dic, 0.5–1.5 μM), purchased from Sigma-Aldrich (Milan, Italy), and harpagoside (HR, 50–100 μM), acquired from Santa Cruz Technology (DBA srl, Milan, Italy), were solubilized in distilled water (dH_2_O) and sterilized by cellulose membrane filter (0.22-µm pore size). The loading of sterilized suspensions (100 μL) to each CaP gel was done in triplicate through a sterile syringe and each drug at different concentrations (Ale, 1 μM; Dic, 0.5–1.5 μM and HR, 50–100 μM) was loaded in 70 mg of CaP-based paste. For enhancing drug adsorption on the CaPs, drug-loaded biomaterials were dried for 2 h at 37 °C (Figure 1). Each drug concentration was chosen according to previous studies that showed the effective concentrations of these drugs in terms of osteogenic or anti-inflammatory activities on in vitro models of osteoporosis [13,19,20]. Here, drugs loaded on the materials were used at different concentrations on the basis of their natural (HR) [9] and synthetic (Ale and Dic) source. Indeed, usually, natural compounds exert therapeutic effects at doses tens or hundreds times higher than of the equivalent synthetic drugs [21].

#### Drug-Release Study

Drug release kinetics from CaPs were determined by the spectrophotometric method. Specifically, drug release kinetic was performed on the compositions having the best osteogenic response and anti-phlogistic activities [CaP plus Diclofenac (1 µM/scaffold) and CaP plus Harpagoside (75 µM/scaffold)]. The CaP systems (70 mg) containing drugs [diclofenac (1 µM/scaffold) and harpagoside (75 µM/scaffold)] were dipped in 1000 µL sterile Tris-buffer solution (pH = 6.8) and kept in a shaking incubator (37 °C, 40 rpm) for time points up to 3 weeks. At designated time points, the supernatant was picked up and an equal amount of fresh medium was added to each sample. Then, 100 μL of supernatant was immediately measured with a UV−vis spectrophotometer (Victor X3 Multilabel Plate Reader, Perkin Elmer, Milan, Italy) at a wavelength of 280 nm in order to detect amino groups and aromatic systems. Here, alendronate release kinetic was not performed because it is a non-chromophoric compound, and its release by basic spectrophotometric methods is not reliable. So, a derivatization reaction would be realized for determining alendronate release by using 2,4-dinitrofluorobenzene (DNFB) as the chromogenic reagent [22].

### 2.2. Biological Tests

#### 2.2.1. Osteoblasts Expansion, Culture, and Proliferation

Human osteoblasts at passages from 2 to 7 were grown in α-modified Eagle’s medium (α-MEM) (Sigma-Aldrich, Milan, Italy) containing 10 vol.% fetal bovine serum (FBS), antibiotic solution (streptomycin 100 µg/mL and penicillin 100 U/mL, Sigma-Aldrich, Italy) and 2 mM L-glutamine in a humidified atmosphere containing 5% CO_2_ at 37 °C. Cell viability (>80%) was evaluated by trypan blue dye. Then, 1 × 10^4^ osteoblasts were re-suspended in medium and were plated onto the materials (V = 150 μL of material for each well). After seeding, materials were maintained in culture for 21 days, with the cell culture medium being changed every 3–4 days. The in vitro cell proliferation was analyzed after 1, 3, 7, 14, and 21 days of culture time by using Alamar blue assay (AbD Serotec, Milan, Italy). AlamarBlue™ solution (10 *v/v* %) was added directly to each well and incubated at 37 °C for 4 h to afford resazurin to resorufin conversion by cells. The optical density was immediately detected using a UV−vis spectrophotometer (Victor X3 Multilabel Plate Reader, Perkin Elmer) at wavelengths of 570 and 600 nm. The Alamar blue assay allows to check the cell growth by measuring the metabolic activity of live cells.

#### 2.2.2. Osteogenic Differentiation Study

The effect of drug-loaded CaP (i.e., alendronate, diclofenac, and harpagoside) on immature osteoblast behavior as osteogenic differentiation in basal medium was analyzed by the expression of early alkaline phosphatase (ALP) and later osteocalcin (OCN) signals of osteogenic differentiation. The phosphatase activity (SensoLyte pNPP ALP assay kit, ANASPEC, Milan, Italy) was analyzed at days 3, 7, 14, and 21 of cell culture in cell lysates (50 µL) by measuring the activity of ALP enzyme after 1 h at 37 °C, as reported in previous studies [23]. The ALP values were adjusted for the number of cells present on each material; indeed, ALP expression was noted as nanograms of ALP normalized to the micrograms of total DNA content detected through a PicoGreen dsDNA quantification kit (Life Technologies, Monza, Italy). Furthermore, osteocalcin (OCN) levels were quantified by using a commercially available kit (Quantikine Human Osteocalcin Immunoassay R&D system, Milan, Italy) according to the manufacturer’s instructions at days 14 and 21 of cell culture.

#### 2.2.3. Scanning Electron Microscopy Analysis for Cell Adhesion Evaluation

Cell–materials interaction in terms of cell attachment was assessed by using Scanning Electron Microscopy (SEM). For SEM analysis, osteoblasts at a density of 2 × 10^4^ were plated onto CaP alone, CaP+Ale (1 μM), CaP+Dic (0.5–1.5 μM), and CaP+HR (75 μM) and were incubated for 3 days at 37 °C. After this time, cells were fixed onto the materials using a solution of 4% paraformaldehyde for 24 h at 4 °C. Later, cell-loaded materials were washed and dehydrated using a series of increasing ethanol concentration. Then, the samples were covered with a thin layer of gold and analyzed in a field-emission scanning electron microscopy (FESEM, QUANTA200, FEI, Eindhoven, The Netherlands) at an accelerating voltage of 30 kV.

#### 2.2.4. In Vitro Anti-Inflammatory Response

For assessing the effect of drug-loaded CaP on cell inflammatory response, basal levels of inflammatory markers were quantified. This effect was examined on experimental in vitro models of inflammation by measuring the basal levels of interleukin 1β (IL-1β) and interleukin-10 (IL-10). For this purpose, osteoblasts were seeded onto samples and in tissue culture 24-well plates (used as control, CTR) at a density of 1 × 10^5^ cells/well. Cell supernatants were utilized to quantify interleukin amount through commercial ELISA kits (Affymetrix, eBioscience Srl, Milan, Italy) as reported on the manufacturer’s instructions. Moreover, for better reproducing the focal bone inflammation, drug-loaded CaP effect in a co-culture system was also evaluated. The co-culture system containing osteoblasts (25,000 cells/well at passages 5–6) and J774 macrophages (50,000 cells/well at passages 25–26) were stimulated after 24 h of culture time by lipopolysaccharide (LPS, 1 μg/mL) and after 3 days of stimulation, IL-1β and IL-10 levels were measured. Morphological features of cell co-cultures were analyzed using an optical microscope (Motic AE31 Inverted Biological Microscope, Seneco srl, Milan, Italy). To this end, cells were fixed with 4% formaldehyde after 3 days of LPS stimulation observed by optical microscope. These outcomes were compared to images of co-cultures seeded on CaP-based materials. These images were acquired using confocal laser scanning microscopy (Leica TCS sp8 confocal microscope, Leica Microsystems Srl, Milan, Italy) after treating co-cultures with cell tracker green for osteoblasts and cell tracker red for macrophages [2]. On experimental in vitro models of inflammation, Dic and HR were used at the concentrations of 1 µM and 75 µM, respectively for their best effects obtained as osteogenic and anti-inflammatory activities in basal condition.

### 2.3. Statistical Analysis

All quantitative experiments were performed in triplicate, and the results were reported as mean ± standard deviation for *n* = 3. Statistical analyses were performed using GraphPad Prism^®^, version 5.00 (GraphPad Software, La Jolla, CA, USA, www.graphpad), and data were compared using a Student’s *t*-test and Kruskal–Wallis test. Values of p less than 0.05 were considered significant.

## 3. Results

### 3.1. Drug Release Study

**Figure 2:**
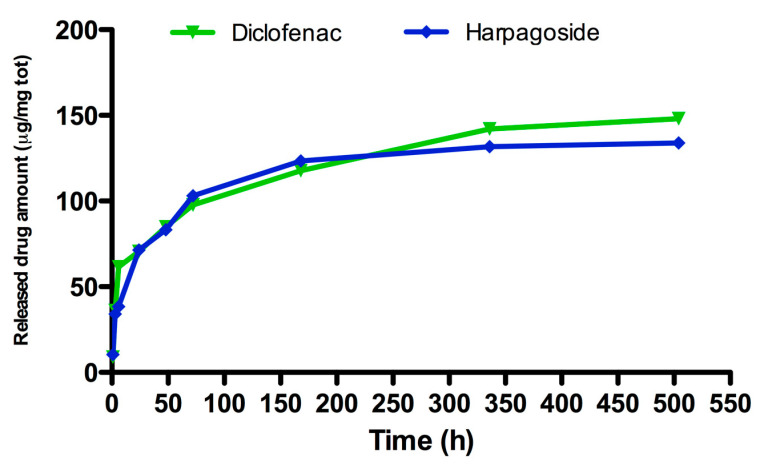
Release study of diclofenac 1 μM and harpagoside (HR) 75 μM from CaP-based materials by using the spectrophotometric method starting from 1 h to 504 h (21 days) of incubation at 37 °C. Data are reported as drug amount released from total milligrams (70 mg) of paste.

Results concerning drug release from injectable CaP carriers have shown comparable release kinetics between diclofenac and harpagoside. Specifically, a burst release in terms of released drug amount was observed for diclofenac-loaded CaP (1 μM) and HR-loaded CaP (75 μM) after 70 h of incubation. Later, HR and diclofenac were released slowly with a constant kinetic until 504 h of incubation (Figure 2). In particular, considering the initial drug amount loaded on CaP of diclofenac (450 μg/70 mg) and HR (370 μg/70 mg), the results revealed that 30–40% of drugs was released after 336 h. After this time point, both drugs were released from CaP in a constant manner up to 504 h monitored.

### 3.2. Effect of Drug-Loaded CaP Based Materials on Cell Proliferation

**Figure 3:**
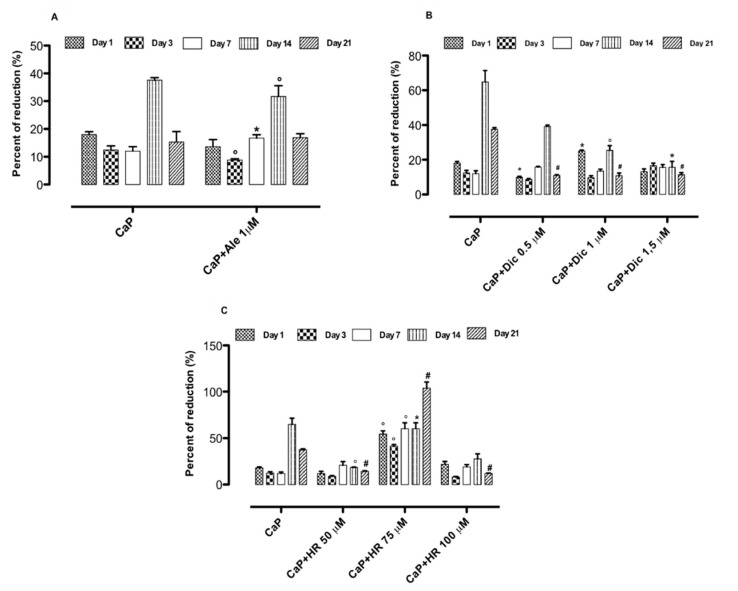
Alamar Blue percent of reduction measured after 1, 3, 7, 14, and 21 days of cell–materials interaction: CaP+Ale (**A**), CaP+Dic (**B**), CaP+HR (**C**). Data represent mean ± dev.st. of 3 independent experiments (*n* = 6). * *p* < 0.05; ° *p* < 0.01; # *p* < 0.001 vs. CaP. Ale: alendronate, Dic: diclofenac sodium, HR: harpagoside.

The biocompatibility of loaded-CaP systems and their control (CaP) was assessed on cell-proliferating behavior. In particular, for the Ale-loaded CaP system, the best result in terms of cell proliferation was obtained at day 14 (Figure 3A). Meanwhile, for the Dic-loaded compositions, the best response concerning cell proliferation was observed for Dic-loaded CaP 0.5 μM at day 14 (Figure 3B), even if CaP alone showed higher values of cell proliferation over culture time. In addition, the biological investigations demonstrated that HR-loaded CaP systems loaded with different concentrations (50–100 μM) determined a good effect on cell proliferation (Figure 3C). In particular, the HR-loaded CaP system with 75 μM significantly increased cell proliferation over culture time, thus suggesting an excellent cell–material interaction (Figure 3C). The HR-loaded CaP system (75 μM) was the only composition that was able to induce higher values in osteoblast proliferation than CaP alone. Cell survival was also evaluated in the presence of drug solutions without CaP to confirm no cytotoxic effects on osteoblasts in the presence of drug solutions at the different concentration used in CaP loading. Figure S1 exhibited no cytotoxic effects of drug solutions at different concentrations used to load injectable CaP carriers. In particular, the HR 75 μM solution showed the best behavior in terms of osteoblast proliferation.

### 3.3. Effect of Drug-Loaded CaP-Based Materials on Osteogenesis

**Figure 4:**
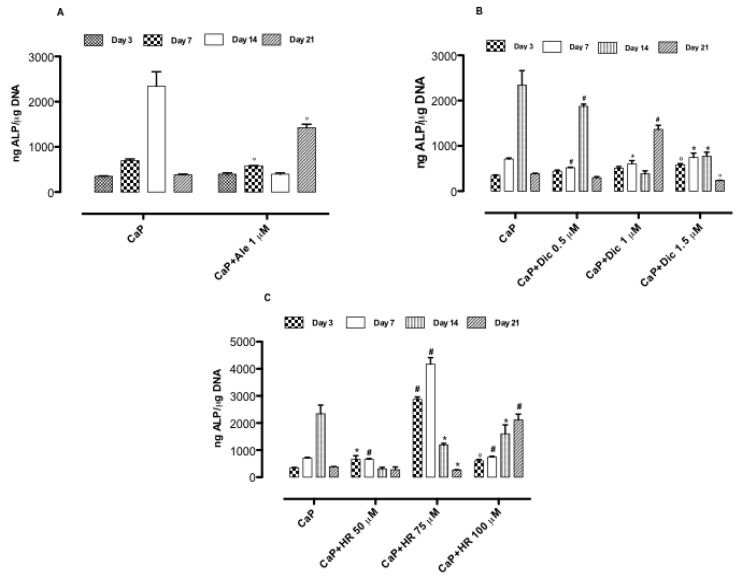
Alkaline phosphatase activity after 3, 7, 14, and 21 days of cell–materials interaction: CaP+Ale (**A**), CaP+Dic (**B**), and CaP+HR (**C**). Data represent mean ± dev.st. of three independent experiments (*n* = 6). * *p* < 0.05; ° *p* < 0.01; # *p* < 0.001 vs. CaP.

**Figure 5:**
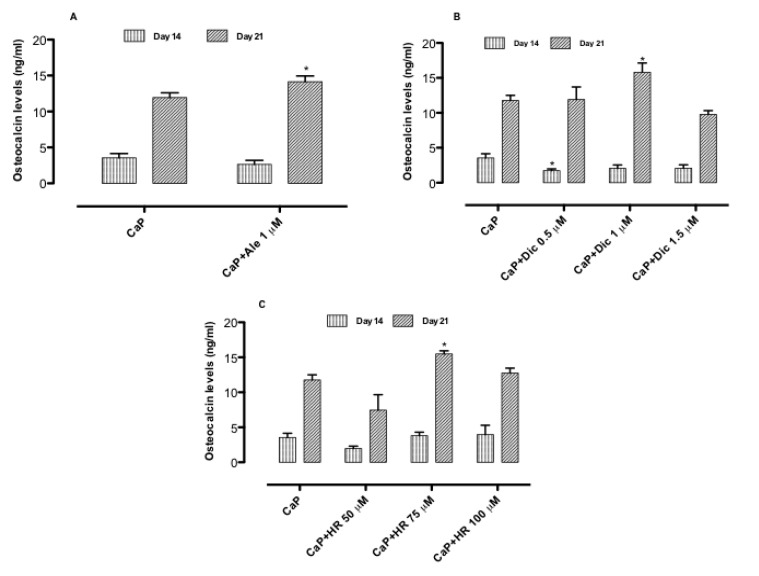
Osteocalcin levels of osteoblasts seeded onto CaP alone and CaP+Ale (**A**), CaP+Dic (**B**), CaP+HR (**C**) in basal medium at days 14 and 21. Data represent mean ± dev.st. of three independent experiments (*n* = 6). * *p* < 0.05 vs. CaP.

The effect of three different compositions on osteogenesis was evaluated through the expression of ALP and OCN, which are early and later markers of differentiation, respectively. The results evidenced that the loading of Ale (1 μM) in the CaP system delays the ALP expression at day 21 compared to the CaP system, where the ALP peak was obtained at day 14 (Figure 4A). Meanwhile, osteocalcin levels were considerably induced by Ale–CaP (1 μM) at day 21 (Figure 4). These results indicated that Ale coupled with CaP allows delaying the CaP osteoinductive effect at long times by maintaining high levels of ALP at day 21 and enhancing OCN expression as a later marker of osteogenesis. For diclofenac-based compositions, the results proved that by increasing diclofenac concentration from 0.5 to 1 μM, a delay in ALP expression was noticed. Indeed, the highest ALP value was obtained for CaP and Dic-loaded CaP (0.5 μM) at day 14 (Figure 4B). By contrast, diclofenac-loaded CaP (1 μM) induced the highest ALP expression at day 21 (Figure 4B), and it significantly increases OCN levels at the same time point (Figure 5B) compared to CaP alone and Dic-loaded CaP (0.5 and 1.5 μM). Concerning an HR-based system, the highest ALP values were detected in the presence of CaP+HR 75 μM at days 3 and 7. The highest values in ALP expression were also induced by HR 75 μM solution without a CaP component after 3 and 7 days of treatment (Figure S2). Conversely, HR100 μM stimulated higher ALP production compared to CaP alone only at day 21 of cell culture (Figure 4C). The highest osteocalcin values were detected in the presence of HR-loaded CaP (75 μM) at day 21 (Figure 4C and Figure 5C). Lower concentrations of HR such as 50 μM did not expose osteoinductive potential, and this inactivity probably is due to the inability to inhibit osteoclastogenesis at lower concentrations [13].

### 3.4. Effect of Drug-Loaded CaP-Based Materials on Basal Inflammatory Response

The biocompatibility of drug-loaded systems was also analyzed as the capability to inhibit inflammatory bone reactions in basal conditions. To this end, the immune response as inflammatory (IL-1β) and anti-inflammatory (IL-10) cytokine production was evaluated. The Ale-loaded CaP showed also a good effect on basal immune response. Indeed, our results indicated that Ale (1 μM), CaP, and Ale-loaded CaP (1 μM) decreased IL-1β levels (pro-inflammatory cytokine) and contemporarily significantly increased IL-10 levels (anti-inflammatory cytokine) at day 3 (Figures S3A and S4A). These effects on interleukin levels suggest a preventive anti-inflammatory effect of Ale-loaded CaP (1 μM). The same effect on basal inflammatory response (Figures S3B and S4B) was obtained for diclofenac solutions at different concentrations (0.5–1.5 μM), CaP, and Dic-loaded CaP (0.5–1.5 μM) systems, where there was a clear increasing of basal IL-10 levels in a concentration-dependent manner (Figure S3B). A beneficial effect on basal immune response, in the presence of HR-loaded CaP, was also observed. Indeed, HR (50–100 μM), CaP, and HR-loaded CaP at different concentrations (50–100 μM) were able to block IL-1β production levels at day 3 (Figure S3C) and increase IL-basal 10 levels (Figure S4C). The highest IL-10 levels were induced by HR-loaded CaP at 50 μM. These results revealed that HR exerts preventive anti-inflammatory beneficial effects at lower concentration (50 μM) than those exhibiting osteoinductive properties (75–100 μM). This behavior may be explained considering the option of different receptors activation directly depending on the different amount of the same drug [24]. For further investigations on anti-inflammatory activity, the compositions that showed the best response in terms of osteoinductive potential (CaP+Ale 1 μM, CaP+Dic 1 μM, and CaP+HR 75 μM) were chosen and tested on osteoporotic inflammation in an in vitro model.

### 3.5. Cell Adhesion on Drugs-Loaded CaP-Based Materials: SEM Analysis

**Figure 6:**
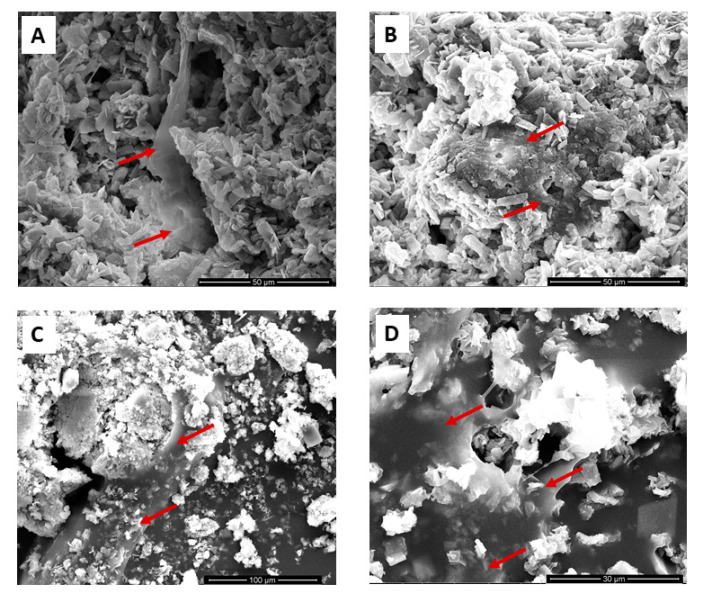
SEM analysis after three days of osteoblast culture onto (**A**) CaP alone, (**B**) CaP+Ale (1 μM), (**C**) CaP+Dic (1 μM), and (**D**) CaP+HR (75 μM). Images are representative of three experiments. Red arrows indicate the presence of cells attached on material surfaces.

SEM images (Figuse 6A–D) revealed that all our compositions analyzed [CaP alone, CaP+Ale (1 μM), CaP+Dic (1 μM) and CaP+HR (75 μM)] promoted good osteoblast adhesion and spreading after three days of cell–materials interaction (Figure 6).

### 3.6. Inflammatory Co-Culture Model

**Figure 7:**
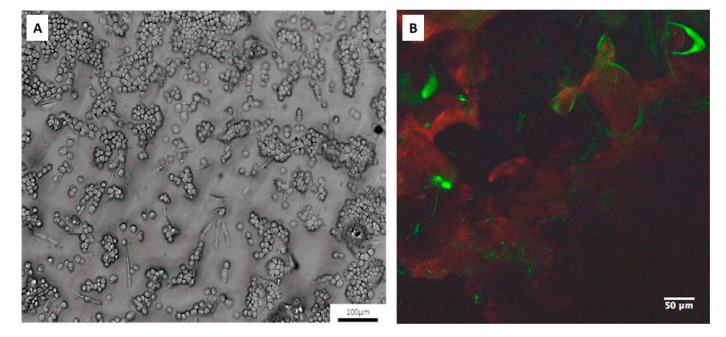
(**A**) Optical (objective 10×) and (**B**) immunofluorescence (objective 40×) analyses on co-cultures containing osteoblasts and macrophages for reproducing in vitro models of bone inflammation.

**Figure 8:**
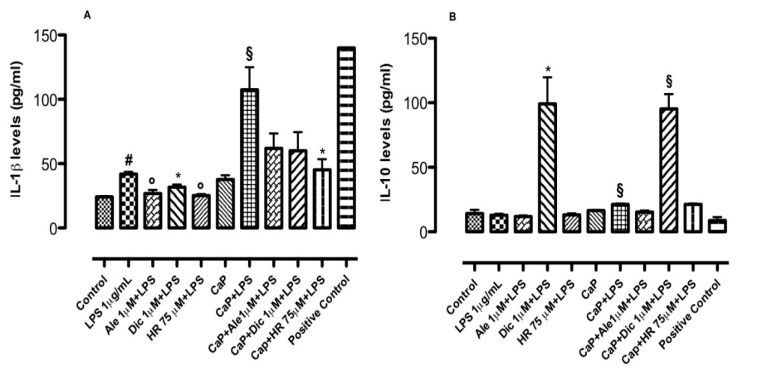
Effect of CaP with and without Ale, Dic, HR, on (**A**) interleukin (IL)-1β and (**B**) IL-10 in co-culture systems (osteoblasts-macrophages) treated with lipopolysaccharide (LPS) (1 μg/mL). Measurements were performed 3 days after LPS (1 μg/mL) stimulation. The exposure of cells to materials with and w/o Ale, Dic, and HR started 24 h before the inflammatory insult. Results (expressed as picograms per mL of supernatant) are mean ± dev.st. of three independent experiments (*n* = 6). (**A**) # *p* < 0.001 vs. control; ° *p* < 0.01 vs. LPS (1 μg/mL); § *p* < 0.01 vs. CaP alone. # *p* < 0.001 vs. control; * *p* < 0.05 vs. LPS (1 μg/mL); § *p* < 0.01 vs. CaP alone and § *p* < 0.01 vs. CaP+LPS (1 μg/mL). # *p* < 0.001 vs. control; ° *p* < 0.01 vs. LPS (1 μg/mL); § *p* < 0.01 vs. CaP alone and * *p* < 0.05 vs. CaP+LPS (1 μg/mL). (**B**) § *p* < 0.01 vs. CaP alone. * *p* < 0.05 vs. LPS (1 μg/mL); § *p* < 0.001 vs. CaP alone and § *p* < 0.001 vs. CaP+LPS (1 μg/mL). § *p* < 0.01 vs. CaP alone.

The anti-inflammatory potential of drug-loaded CaP materials was also evaluated by an in vitro model of bone inflammation consisting of osteoblasts–macrophages co-culture on CaP materials (Figure 7B) stimulated by LPS, which is a well-known inflammatory endotoxin. The images obtained through confocal analysis regarding cell co-culture grown on CaP injectable materials confirmed the coexistence of osteoblasts (green signal) and macrophages aggregates (red signal) as observed in the optical images used as plate control (Figure 7B). On this in vitro model, inflammatory response regarding cytokines production was detected. Alendronate and diclofenac sodium at concentrations of 1 μM were chosen to explore the effect on bone inflammatory response for their best behavior showed on osteogenesis and inflammation in basal conditions. The results demonstrated that Ale-loaded CaP (1 μM) reduced IL-1β levels induced by LPS (Figure 8A). By contrast, it was not capable to increase IL-10 levels (Figure 8B). Conversely, Dic-loaded CaP (1 μM) significantly decreased IL-1β levels induced by LPS (1 μg/mL) at day 3 (Figure 8A) and significantly increased IL-10 levels (Figure 7B). In line with osteogenic effects, the concentration of 75 μM has been chosen to deepen the effect of HR-loaded CaP on the in vitro model of bone inflammation. At this concentration, the system decreased IL-1β levels induced by LPS at day 3 (Figure 8A), even if any changes were observed for IL-10 levels (Figure 8B). The analysis of variance performed using the Kruskal–Wallis test revealed that the medians vary significantly both for IL-1β (*p* value < 0.0001) and IL-10 (*p* value < 0.002) results.

## 4. Discussion

Bone defects concern the worldwide population and constitute a public health problem. Principally, bone fractures deriving from bone disorders are the primary cause of morbidity and disability for the elderly population, thus decreasing life quality and rising mortality [25]. For instance, it was estimated that in the USA, two million people are exposed to osteoporosis-derived fractures every year with an economic burden of nearly $20 billion [26,27]. Hence, it is required to find novel approaches for bone defects treatment. In the area of regenerative medicine, much attention has been devoted to the application of nanostructured biomaterials loaded with stem cells and bone precursors for promoting native bone architecture reconstruction [28,29,30,31]. Recent approaches are aimed to synthetize biomaterials that mimic native bone. In this scenario, injectable calcium phosphate (CaP) is receiving a special interest as a carrier of drugs or osteoinductive compounds (i.e., growth factors) thanks their analogy to bone inorganic component. Several findings reported the possibility to develop CaP-based materials by using different technologies. Specifically, the sol–gel method has a great potential in performing injectable CaP-based biomaterials for bone defect repair because it allows to work at lower temperatures, thus offering the chance to include in the materials a broad spectrum of therapeutic agents [14] and also allows to obtain a crystal length of about 50–100 nm, which is important for protein and drug adsorption. Here, we approached the association of CaP with different natural and synthetic drugs, which promote bone regeneration and inhibit local inflammatory response. Alendronate is the most famous bisphosphonate applied for the control of postmenopausal and glucocorticoids-induced osteoporosis thanks to its ability to reduce fracture risk [32]. However, orally administered alendronate causes esophageal ulceration, stomach pain, acid reflux, constipation, diarrhea, upset stomach, nausea, muscle, and joint pain [33]. To improve alendronate compliance and effectiveness, here, we suggest a local administration of this potent biphosphonate combined with CaP as bone component precursors. The same investigations performed for alendronate-loaded CaP were carried out also in presence of diclofenac-loaded CaP at different concentrations (0.5–1.5 μM). It is well known that non-steroidal anti-inflammatory drugs (NSAIDs) are extensively used in patients affected by vertebral osteoporotic fractures. Indeed, osteoporosis occurs with a chronic pain symptomatology [34], such as to require the use of analgesics and/or non-steroidal anti-inflammatory drugs (NSAIDs). Additionally, patients with osteoporosis are mostly older and often suffer from a rheumatic comorbidity, thus feeling the necessity for a combined therapy, since they must often also take specific pharmacological therapy for osteoporosis. This may entail difficulties in maintaining compliance or worsening the risk of adverse reactions, especially GI dysfunctions [35]. On the other hand, there are many studies showing that the administration of NSAIDs would have a protective effect against bone loss [36]. To reduce side effects related to a combined treatment, in this work, we approached the loading of diclofenac, one of the most famous NSAIDs, in CaP-injectable materials for a local treatment of osteoporotic bone loss and fractures. Furthermore, taking into consideration the inflammatory microenvironment existing during osteoporosis process, a good bone substitute should modulate the inflammatory reaction in the injection site [2]. Hence, it was interesting to analyze the effect of alendronate-loaded CaP on inflammatory response on osteoblasts in basal conditions. Besides the use of conventional synthetic drugs, the pharmacological research is always looking for innovative natural compounds and functional molecules useful for the therapy of various pathologies. In this scenario, it was estimated that iridoids glycosides display an encouraging anti-inflammatory potential. Moreover, iridoids are famous in traditional medicine for their therapeutic activities including cardiovascular, hepatoprotection, hypoglycaemic, antimutagenic, antispasmodic, anti-tumor, antiviral, immunomodulation, and purgative effects [37]. In particular, harpagoside (HR), a compound isolated from H. procumbens, exhibited a strong anti-inflammatory activity in inhibiting LPS induced Prostaglandin E_2_ (PGE2) synthesis and nitric oxide production. Furthermore, some authors showed that HR exerts analgesic effects on an in vivo model of osteoarthritis [38]. However, the mechanism of action for HR has not yet clearly elucidated [36]. A recent research has found that HR blocks receptor activator of nuclear factor κB ligand (RANKL)-induced osteoclastogenesis on in vitro studies and suppresses inflammation-induced bone loss on in vivo mouse models. Indeed, HR impedes the formation of osteoclasts from mouse bone marrow macrophages (BMMs) as well as LPS-induced bone loss in an inflammatory osteoporosis model but does not limit ovariectomy-mediated bone erosion [13]. According to these previous data, here, we have realized HR-loaded CaP materials, and their effects on osteoblast proliferation, differentiation, and immune response were investigated. As in this work, previous strategies based on localized drug delivery on in vitro bone inflammation models have been attempted [20]. A system consisting of diclofenac sodium loaded onto poly(D,L-lactic acid-co-glycolic acid)/poly(ethylene glycol) scaffolds decreased inflammation on an in vitro osteoblast model. However, these scaffolds loaded with diclofenac induced a biological response in terms of osteogenesis in the presence of conditioned osteogenic medium [20]. Our findings proved that the association of hydroxyapatite precursors (CaP) and Diclofenac (CaP+Dic 0.5 or 1 μM) induced high levels of ALP and OCN after 14 and 21 days of cell culture, respectively, in basal condition without adding external osteoinductive components. A different behavior the in presence of the same composition (CaP) conjugated with a biphosphonate (CaP+Alendronate 1 μM) was observed. Indeed, a delay in terms of ALP expression (21 days) induced by CaP+Alendronate (1 μM) compared to CaP alone and other similar systems, which have been previously explored, was observed [39]. Indeed, alendronate released from biphasic calcium phosphate (BCP) scaffolds increased ALP expression in MG-63 cells after about 7 and 10 days of cell culture [39]. Despite this sustained ALP expression, the system (CaP+Alendronate 1 μM) showed anti-inflammatory properties on an in vitro model of bone inflammation, thus decreasing inflammatory marker expression. Not much is known on biomaterials functionalized with harpagoside for the localized treatments of bone disorders. Our approach consisting in the design and study of HR-loaded CaP compositions (natural anti-inflammatory drug) on in vitro bone models revealed that the combination of CaP with HR 75 μM possesses remarkable osteoinductive properties (earlier ALP expression), thus suggesting the effectiveness to combine anti-inflammatory natural drugs and bone precursors in the induction of osteogenesis. Furthermore, this assertion was validated by high values of OCN, as a famous marker of osteogenesis involved in later phases of bone formation and useful as an index of mineralization induction processes. The highest OCN values obtained in the presence of CaP+Dic 1 μM and CaP+HR 75 μM compositions, after 14 days of cell culture, are in line with the results observed in drug release kinetics, where a plateau in released drug amount was properly reached at day 14. In the area of biomaterials for bone regeneration, the gold standard is to develop devices with both osteoinductive and anti-inflammatory properties because to prevent and treat bone inflammation enhances bone turnover by blocking osteoclastogenesis [2]. Here, we tested the capability of drug-loaded systems to prevent (basal conditions) and treat (LPS stimulated co-culture) the inflammatory response. Thus, our data revealed that all drug-loaded CaP materials inhibit IL-1β, which is a well-known pro-inflammatory cytokine, secretion in basal conditions. High levels induced by drug-loaded CaP materials were observed also in the case of IL-10 cytokine involved in anti-inflammatory response, but significant IL-10 values were obtained only in the presence of CaP+Dic 1 μM. These results are in line with the certified anti-inflammatory activity of diclofenac. Furthermore, these data fit in with the burst release of Dic 1 μM and HR 75 μM after 70 h ascribable to ant-inflammatory activity appearance. The mechanism of action for CaP+HR 75 μM on inflammatory response prevention is unknown, but it is probably though the activation of some receptor involved in anti-inflammatory response and related to a specific concentration of HR. Lastly, the capability of our drug-loaded materials to act also in an inflamed microenvironment was analyzed using a co-culture in vitro model stimulated by a flogistic agent (LPS), as previously described. In inflammatory conditions, all the compositions at specific concentration (Ale 1 μM, Dic 1 μM and HR 75 μM) explained an inhibitory effect on IL-1β induced by LPS. By contrast, only Dic 1 μM induced anti-inflammatory mechanisms activation by increasing IL-10 levels after a phlogistic stimulus.

## 5. Conclusions

The focus of this study was to realize injectable drug-loaded systems for bone defects in order to inhibit bone degeneration and inflammatory activities with the subsequent enhancement of new-forming bone. Our findings on osteoinductive and anti-inflammatory properties of injectable drug-loaded systems obtained combining bone precursors (CaP) and drugs with different activities (alendronate, diclofenac, and harpagoside) demonstrated that the materials may be good potential candidates that are able to improve bone disorders therapy by treating simultaneously bone inflammation and resorption. In conclusion, the three systems (alendronate-loaded CaP, diclofenac-loaded CaP, and HR-loaded CaP) exert beneficial osteogenic and anti-inflammatory activities in vitro. Thus, the proposed systems represent a future, potential multi-target strategy for the local therapy of inflammation and bone loss determined by osteoporosis.

## Supplementary Materials

The following are available online at https://www.mdpi.com/2079-4991/10/9/1743/s1, Further details on Drug-loaded CaP based materials. Figure S1: Alamar Blue percent of reduction measured at days 1, 3, 7, 14 and 21 of osteoblasts exposure to drug solutions. Data represent mean ± dev.st. of 3 independent experiments (*n* = 6). * *p* < 0.05; ° *p* < 0.01; # *p* < 0.001 vs. Control; Figure S2: Alkaline phosphatase activity after 3, 7, 14, 21 days of exposure of osteoblast cell cultures to drug solutions. Data represent mean ± dev.st. of 3 independent experiments (*n* = 6). * *p* < 0.05; ° *p* < 0.01; # *p* < 0.001 vs. Control; Figure S3: Effect of CaP with and w/o Ale (A), Dic (B), HR (C) on basal IL-1β on osteoblast cell cultures after 72 h. Results, expressed as interleukin levels (pg/mL), are mean ± dev.st. of 3 independent experiments (*n* = 6). (A) § *p* < 0.05, ° *p* < 0.01 and § *p* < 0.001 vs. positive control. (B) * *p* < 0.05, ° *p* < 0.01, # *p* < 0.001 vs. control; * and § *p* < 0.05, ° *p* < 0.01 and § *p* < 0.001 vs. positive control. (C) * *p* < 0.05, ° *p* < 0.01, # *p* < 0.001 vs. control; * and § *p* < 0.05, ° *p* < 0.01 and § *p* < 0.001 vs. positive control; Figure S4: Effect of CaP with and w/o Ale (A), Dic (B), HR (C) on basal IL-10 on osteoblast cell cultures after 72 h. Results, expressed as interleukin levels (pg/mL), are mean ± dev.st. of 3 independent experiments (*n* = 6). (A) § *p* < 0.05, ° *p* < 0.01 and § *p* < 0.001 vs. positive control. (B) * *p* < 0.05, ° *p* < 0.01, # *p* < 0.001 vs. control; * and § *p* < 0.05, ° *p* < 0.01 and § *p* < 0.001 vs. positive control. (C) * *p* < 0.05, ° *p* < 0.01, # *p* < 0.001 vs. control; * and § *p* < 0.05, ° *p* < 0.01 and § *p* < 0.001 vs. positive control.

## References

[1] Alt V., Thormann U., Ray S., Zahner D., Dürselen L., Lips K., El Khassawna T., Heiss C., Riedrich A., Schlewitz G. (2013). A new metaphyseal bone defect model in osteoporotic rats to study biomaterials for the enhancement of bone healing in osteoporotic fractures. Acta Biomater..

[2] Fasolino I., Raucci M.G., Soriente A., Demitri C., Madaghiele M., Sannino A., Ambrosio L. (2019). Osteoinductive and anti-inflammatory properties of chitosan-based scaffolds for bone regeneration. Mater. Sci. Eng. C.

[3] Curtis E.M., Moon R.J., Dennison E., Harvey N.C., Cooper C. (2015). Recent advances in the pathogenesis and treatment of osteoporosis. Clin. Med..

[4] Thormann U., Ray S., Sommer U., Sommer U., ElKhassawna T., Rehling T., Hundgeburth M., Henß A., Rohnke M., Janek J. (2013). Bone formation induced by strontium modified calcium phosphate cement in critical-size metaphys e al fracture defects in ovariectomized rats. Biomaterials.

[5] Kennel K.A., Drake M.T. (2009). Adverse effects of bisphosphonates: Implications for osteoporosis management. Mayo Clin. Proc..

[6] Raucci M.G., Álvarez-Pérez M.A., Meikle S.T., Ambrosio L., Santin M. (2014). Poly(Epsilon-Lysine) Dendrons Tethered with Phosphoserine Increase Mesenchymal Stem Cell Differentiation Potential of Calcium Phosphate Gels. Tissue Eng. Part A.

[7] Low K.L., Tan S.H., Zein S.H.S., Roether J.A., Mouriño V., Boccaccini A.R. (2010). Calcium phosphate-based composites as injectable bone substitute materials: A review. J. Biomed. Mater. Res. Part B Appl. Biomater..

[8] Mncwangi N., Chen W., Vermaak I., Viljoen A., Gericke N. (2012). Devil’s Claw—A review of the ethnobotany, phytochemistry and biological activity of Harpagophytum procumbens. J. Ethnopharmacol..

[9] Denner S.S. (2007). A Review of the Efficacy and Safety of Devilʼs Claw for Pain Associated With Degenerative Musculoskeletal Diseases, Rheumatoid, and Osteoarthritis. Holist. Nurs. Pract..

[10] Anauate M.C., Torres L.M., De Mello S.B.V. (2010). Effect of isolated fractions of Harpagophytum procumbens D.C. (devil’s claw) on COX-1, COX-2 activity and nitric oxide production on whole-blood assay. Phytother. Res..

[11] Inaba K., Murata K., Naruto S., Matsuda H. (2010). Inhibitory effects of devil’s claw (secondary root of Harpagophytum procumbens) extract and harpagoside on cytokine production in mouse macrophages. J. Nat. Med..

[12] Haseeb A., Chen D., Haqqi T.M. (2013). Delphinidin inhibits IL-1beta-induced activation of NF-kappaB by modulating the phosphorylation of IRAK-1(Ser376) in human articular chondrocytes. Rheumatology.

[13] Kim J.-Y., Park S.-H., Baek J.M., Erkhembaatar M., Kim M.S., Yoon K.-H., Oh J., Lee M.S. (2015). Harpagoside Inhibits RANKL-Induced Osteoclastogenesis via Syk-Btk-PLCγ2-Ca2+Signaling Pathway and Prevents Inflammation-Mediated Bone Loss. J. Nat. Prod..

[14] Raucci M.G., Fasolino I., Pastore S.G., Soriente A., Capeletti L.B., Dessuy M.B., Giannini C., Schrekker H.S., Ambrosio L. (2018). Antimicrobial Imidazolium Ionic Liquids for the Development of Minimal Invasive Calcium Phosphate-Based Bionanocomposites. ACS Appl. Mater. Interfaces.

[15] Prieto E.M., Page J.M., Harmata A.J., Guelcher S.A. (2013). Injectable foams for regenerative medicine. Wiley Interdiscip. Rev. Nanomed. Nanobiotechnol..

[16] D’Antò V., Raucci M.G., Guarino V., Martina S., Valletta R., Ambrosio L. (2013). Behaviour of human mesenchymal stem cells on chemically synthesized HA-PCL scaffolds for hard tissue regeneration. J. Tissue Eng. Regen. Med..

[17] Raucci M.G., Giugliano D., Longo A., Zeppetelli S., Carotenuto G., Ambrosio L. (2016). Comparative facile methods for preparing graphene oxide-hydroxyapatite for bone tissue engineering. J. Tissue Eng. Regen. Med..

[18] Dessì M., Raucci M.G., Zeppetelli S., Ambrosio L. (2012). Design of injectable organic-inorganic hybrid for bone tissue repair. J. Biomed. Mater. Res. Part A.

[19] Kim H.K., Kim J.H., Abbas A.A., Yoon T.R. (2008). Alendronate Enhances Osteogenic Differentiation of Bone Marrow Stromal Cells: A Preliminary Study. Clin. Orthop. Relat. Res..

[20] Sidney L.E., Heathman T.R., Britchford E.R., Abed A., Rahman C.V., Buttery L.D. (2015). Investigation of localized delivery of diclofenac sodium from poly(D,L-lactic acid-co-glycolic acid)/poly(ethylene glycol) scaffolds using an in vitro osteoblast inflammation model. Tissue Eng. Part A.

[21] Karimi A., Majlesi M., Rafieian-Kopaei M. (2015). Herbal versus synthetic drugs; beliefs and facts. J. Nephronpharmacol..

[22] Walash M.I., Metwally M.E.-S., Eid M.I., El-Shaheny R. (2012). Validated spectrophotometric methods for determination of Alendronate sodium in tablets through nucleophilic aromatic substitution reactions. Chem. Cent. J..

[23] Raucci M.G., Fasolino I., Caporali M., Serrano-Ruiz M., Soriente A., Peruzzini M., Ambrosio L. (2019). Exfoliated Black Phosphorus Promotes in Vitro Bone Regeneration and Suppresses Osteosarcoma Progression through Cancer-Related Inflammation Inhibition. ACS Appl. Mater. Interfaces.

[24] Lowe E.S., Balis F.M. (2007). Principles of Clinical Pharmacology.

[25] Borgström F., Lekander I., Ivergård M., Strom O., Svedbom A., Alekna V., Bianchi M.L., Clark P., Curiel M.D., Dimai H.P. (2013). The International Costs and Utilities Related to Osteoporotic Fractures Study (ICUROS)—Quality of life during the first 4 months after fracture. Osteoporos. Int..

[26] Burge R., Dawson-Hughes B., Solomon D.H., Wong J.B., King A., Tosteson A. (2006). Incidence and Economic Burden of Osteoporosis-Related Fractures in the United States, 2005–2025. J. Bone Miner. Res..

[27] Ensrud K.E. (2013). Epidemiology of Fracture Risk With Advancing Age. J. Gerontol. Ser. A Boil. Sci. Med Sci..

[28] Amini A.R., Laurencin C.T., Nukavarapu S. (2012). Bone tissue engineering: Recent advances and challenges. Crit. Rev. Biomed. Eng..

[29] O’Keefe R.J., Mao J.J. (2011). Bone Tissue Engineering and Regeneration: From Discovery to the Clinic—An Overview. Tissue Eng. Part B Rev..

[30] Ducheyne P., Mauck R.L., Smith U.H. (2012). Biomaterials in the repair of sports injuries. Nat. Mater..

[31] Steinert A.F., Rackwitz L., Gilbert F., Nöth U., Tuan R.S. (2012). Concise Review: The Clinical Application of Mesenchymal Stem Cells for Musculoskeletal Regeneration: Current Status and Perspectives. Stem Cells Transl. Med..

[32] Prinsloo P.J.J., Hosking D.J. (2006). Alendronate Sodium in the Management of Osteoporosis. Ther. Clin. Risk Manag..

[33] Tóth E., Fork F.T., Lindelöw K., Lindström E., Verbaan H., Veress B. (1998). Alendronate-induced severe esophagitis. A rare and severe reversible side-effect illustrated by three case reports. Lakartidningen.

[34] Rossini M., Bertoldo F., Lovato R., Bortolotti R., Gatti D., Adami S. (2003). Use of nonsteroidal anti-inflammatory drugs in patients with vertebral osteoporotic fractures. Reumatismo.

[35] Cappell M.S., Schein J.R. (2000). Diagnosis and treatment of nonsteroidal anti-inflammatory drug-associated uppergastrointestinal toxicity. Gastroenterol. Clin. N. Am..

[36] Graham D.Y., Malaty H.M. (2001). Alendronate and naproxenare synergistic for development of gastric ulcers. ArchIntern. Med..

[37] Viljoen A., Mncwangi N., Vermaak I. (2012). Anti-Inflammatory Iridoids of Botanical Origin. Curr. Med. Chem..

[38] Andersen M.L., Santos E.H., Seabra M.D.L.V., Da Silva A.A., Tufik S. (2004). Evaluation of acute and chronic treatments with Harpagophytum procumbens on Freund’s adjuvant-induced arthritis in rats. J. Ethnopharmacol..

[39] Park K.-W., Yun Y.-P., Kim H.J., Song H.-R. (2015). The Effect of Alendronate Loaded Biphasic Calcium Phosphate Scaffolds on Bone Regeneration in a Rat Tibial Defect Model. Int. J. Mol. Sci..

