# Supplementary material for: Osteogenic and Anti-Inflammatory Behavior of Injectable Calcium Phosphate Loaded with Therapeutic Drugs
Source: Nanomaterials (Basel). 2020 Sep 3;10(9):1743. doi: 10.3390/nano10091743 (PMC7558013; doi:10.3390/nano10091743)
Supplement: Supplementary file 1 [file nanomaterials-10-01743-s001.pdf]

*Supplementary Materials*

# Osteogenic and Anti-Inflammatory Behavior of Injectable Calcium Phosphate Loaded with Therapeutic Drugs

**Ines Fasolino *, Alessandra Soriente, Luigi Ambrosio and Maria Grazia Raucci ***

Institute of Polymers, Composites and Biomaterials—National Research Council (IPCB-CNR), Mostra d'Oltremare pad.20—Viale J.F. Kennedy 54, 80125 Naples, Italy; alessandra.soriente@cnr.it (A.S.); luigi.ambrosio@cnr.it (L.A.)

* Correspondence: ines.fasolino@cnr.it (I.F.); mariagrazia.raucci@cnr.it (M.G.R.)

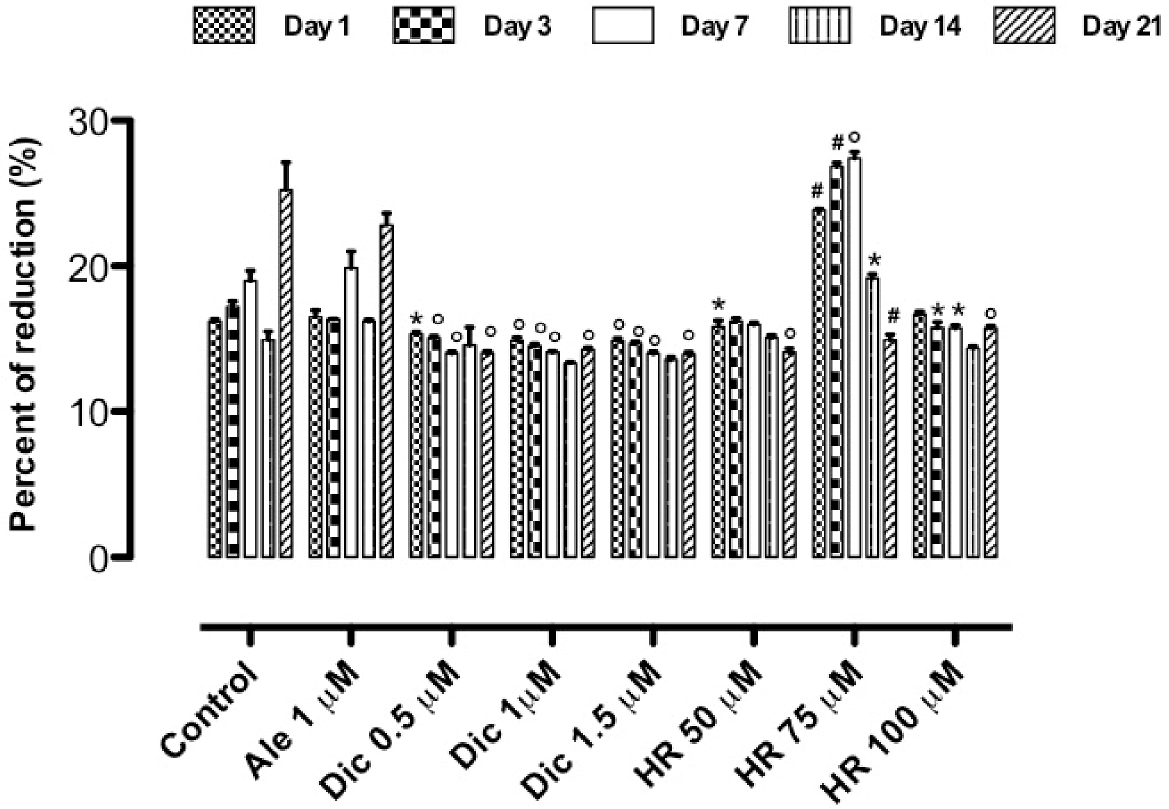


**Figure S1.** Alamar Blue percent of reduction measured at days 1, 3, 7, 14 and 21 of osteoblasts exposure to drug solutions. Data represent mean ± dev.st. of 3 independent experiments ($n$ = 6). * $p < 0.05$; ° $p < 0.01$; # $p < 0.001$ vs. Control.

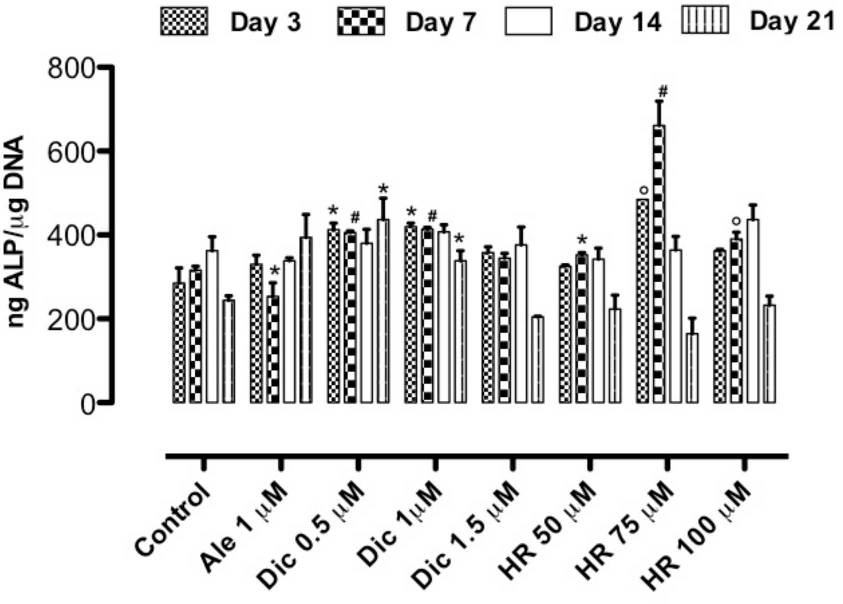


**Figure S2.** Alkaline phosphatase activity after 3, 7, 14, 21 days of exposure of osteoblast cell cultures to drug solutions. Data represent mean ± dev.st. of 3 independent experiments (*n* = 6). * $p < 0.05$; ° $p < 0.01$; # $p < 0.001$ vs. Control.

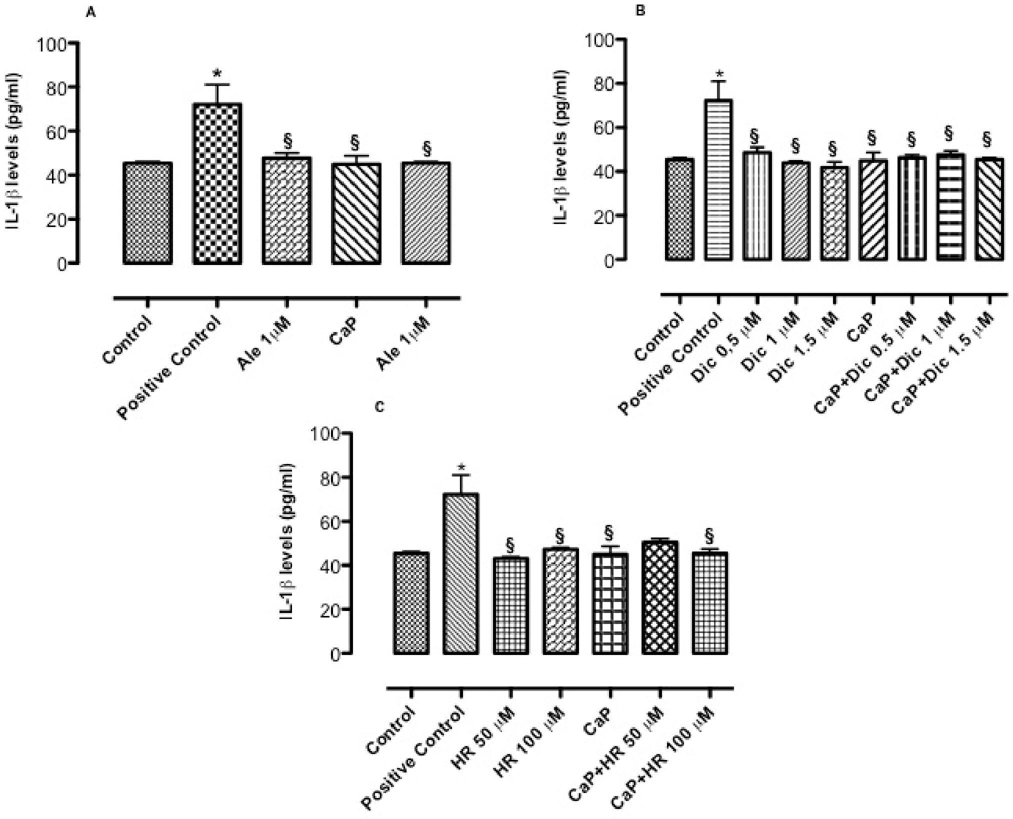


**Figure S3.** Effect of CaP with and w/o Ale (**A**), Dic (**B**), HR (**C**) on basal IL-1β on osteoblast cell cultures after 72 h. Results, expressed as interleukin levels (pg/mL), are mean ± dev.st. of 3 independent experiments (*n* = 6). (A) § $p < 0.05$, ° $p < 0.01$ and § $p < 0.001$ vs. positive control. (B) * $p < 0.05$, ° $p < 0.01$, # $p < 0.001$ vs. control; * and § $p < 0.05$, ° $p < 0.01$ and § $p < 0.001$ vs. positive control. (C) * $p < 0.05$, ° $p < 0.01$, # $p < 0.001$ vs. control; * and § $p < 0.05$, ° $p < 0.01$ and § $p < 0.001$ vs. positive control.

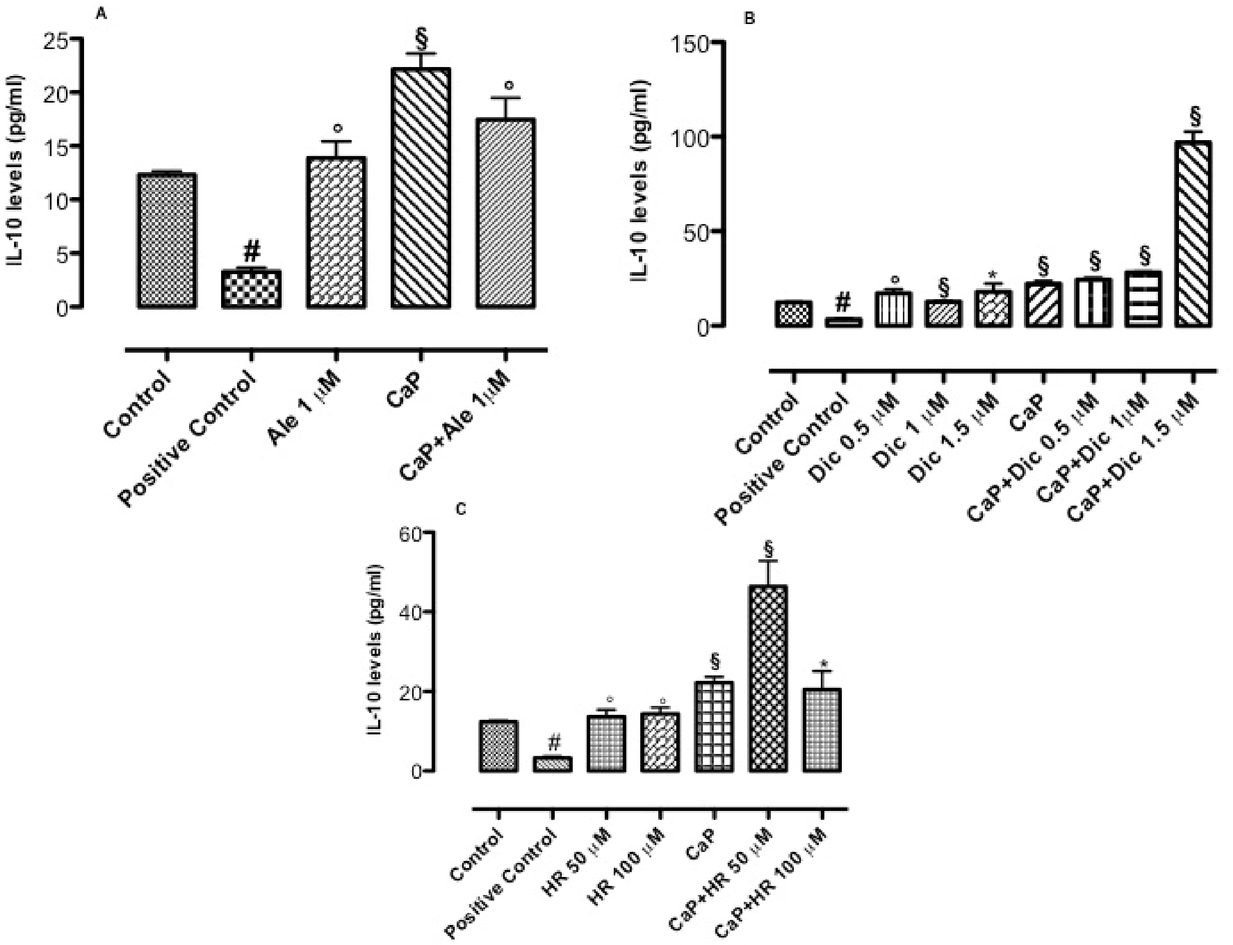


**Figure S4.** Effect of CaP with and w/o Ale (**A**), Dic (**B**), HR (**C**) on basal IL-10 on osteoblast cell cultures after 72 h. Results, expressed as interleukin levels (pg/mL), are mean ± dev.st. of 3 independent experiments ($n$ = 6). (A) § $p < 0.05$, ° $p < 0.01$ and § $p < 0.001$ vs. positive control. (B) * $p < 0.05$, ° $p < 0.01$, # $p < 0.001$ vs. control; * and § $p < 0.05$, ° $p < 0.01$ and § $p < 0.001$ vs. positive control. (C) * $p < 0.05$, ° $p < 0.01$, # $p < 0.001$ vs. control; * and § $p < 0.05$, ° $p < 0.01$ and § $p < 0.001$ vs. positive control.
